# Unlocking Phytoplankton Metallomes with Comparative Analysis of Metal Quotas, Quantitative Proteomics, and Inferred Metalloproteomes

**DOI:** 10.1021/acs.est.5c11233

**Published:** 2025-12-12

**Authors:** Qiong Zhang, Jiayou Ge, Fengjie Liu, Shabaz Mohammed, Kedong Yin, Rosalind E. M. Rickaby

**Affiliations:** † Department of Ocean Science, 58207 The Hong Kong University of Science and Technology, Clear Water Bay, Kowloon, Hong Kong SAR 999077, China; ‡ Center for Ocean Research in Hong Kong and Macau (CORE), The Hong Kong University of Science and Technology, Clear Water Bay, Kowloon, Hong Kong SAR 999077, China; § 26469 Southern Marine Science and Engineering Guangdong Laboratory (Zhuhai), Zhuhai 519000, China; ∥ Grantham Institute-Climate Change and the Environment and Department of Life Sciences, 4615 Imperial College London, Exhitition Road, London SW7 2AZ, United Kingdom; ⊥ Department of Biochemistry, 6396 University of Oxford, South Parks Road, Oxford OX1 3QU, United Kingdom; # Department of Chemistry, University of Oxford, South Parks Road, Oxford OX1 3TA, United Kingdom; ∇ School of Marine Science, Sun Yat-Sen University, Zhuhai 519000, China; ○ Department of Earth Sciences, University of Oxford, South Parks Road, Oxford OX1 3AN, United Kingdom

**Keywords:** trace metals, metalloproteins, phytoplankton, environmental adaptation

## Abstract

Metalloproteins, which can bind with one or more metals, are the basis of many important biological processes in a marine environment. The metalloproteome data for phytoplankton are limited, hindering our understanding of trace metal requirements and their biological function in these primary producers. Here, we conducted a semiquantitative analysis of metal requirements using a protein modeling approach across several phytoplankton species, including the chlorophytes *Ostreococcus tauri* and *Chlamydomonas reinhardtii*, the haptophyte *Geophyracapsa huxleyi*, and the cyanobacterium *Synechocystis*. Our results show strong alignment between trace metal requirements inferred from the proteome and those measured by ICP-MS, with the metalloproteome providing deeper insights into the biological roles of each metal compared with ICP-MS, which indicates only cellular metal abundance. Among all metalloproteins, those containing Mg are the most abundant in all phytoplankton studied here. Among the trace elements, Zn, Fe, and Mn are the most abundant cofactors found in phytoplankton proteins. The cyanobacterium has a much higher percentage of Fe in its expressed proteome compared to the eukaryotes studied here, agreeing with findings from previous comparative genomic studies that trace element requirements are different in prokaryotic and eukaryotic phytoplankton. Using *G. huxleyi* strains from distinct oceanic environments, we further demonstrated that their metalloproteome can be used to identify limiting metals and understand the strategies that phytoplankton use to adapt to specific environments. These findings enhance our understanding of the interactions between biota and their metal environments.

## Graphical abstract

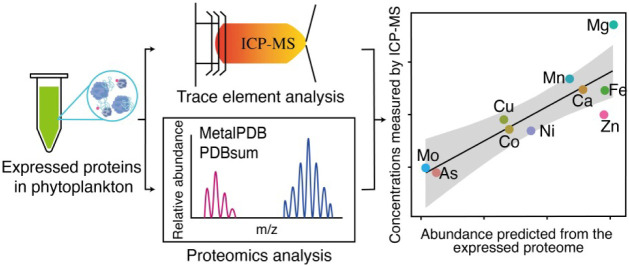

## Introduction

Many important biological processes of phytoplankton, including photosynthesis, carbon fixation, and nitrogen reduction, rely on enzymes that have metals as cofactors.^1^ It has been estimated that half of all enzymes must be associated with a particular metal to function.^2^ The availability of metals in the environment has changed over geological time and, given imperfections in the selectivity of metal transport systems, proteins have evolved to make the best use of metals that are more available.^3–5^ Depending on their evolutionary histories, different groups of marine phytoplankton may have differences in their metalloprotein compositions and metal transport strategies. The evolving trace metal availability in the environment, thus, may provide a selective pressure to promote the proliferation of different phytoplankton groups in the oceanic environment, affecting the phytoplankton community structure with knock-on effects on the marine food web and carbon sequestration.^5^ It is crucial to understand the utilization of trace metals by different groups of marine phytoplankton that inhabit contrasting niches in the modern ocean, not only by assessing the accumulation of trace metals in cells but also examining the functions of trace metals in different biological pathways.

In previous studies, the utilization or requirements of trace metals by marine phytoplankton were mostly determined by measuring the elemental stoichiometry in cells (metallome or quota of trace metals).^6–8^ In the field, attempts have also been made to investigate the elements in phytoplankton^9,10^ which have then been compared with profiles in the abiotic environment to understand their relationships.^11^ The discovery of various metal storage proteins and the plasticity in elemental quotas under different environmental conditions raise the question of whether intracellular trace metal quotas reflect true differences in trace metal usage by phytoplankton.^12,13^ In the past few decades, more and more studies have focused on metal–protein partnerships, using approaches such as genome-wide analysis to identify homologues of known metalloproteins and other deduced metal-binding motifs encoded within sequenced genomes.^5,14^ However, this approach has limitations in estimating metal abundance in cells because not all genes predicted in the genome would be translated and expressed as proteins under all environments, and a single gene for a metalloprotein could be highly expressed, such that a translation from genetic presence to a required concentration is not linear and could be environment-dependent. To obtain *in situ* metal abundance in metalloproteins in cells, native separation of proteins by HPLC or PAGE followed by the detection of metals using ICP-MS^15,16^ has been used. This approach also has limitations in that, without bioinformatics, it cannot identify the function of proteins within which the metals are found, and it may also depend on the composition of the media to which the organism has been exposed and in which it has been growing. An ideal way to solve the problem would be to separate and purify each of the proteins and then identify both the associated metal (*e.g.,* by ICP-MS) and the protein sequence (*e.g.,* by mass spectrometry). However, this approach is constrained by the resolution of chromatographic or electrophoretic techniques, making it challenging to establish the metal–protein associations unambiguously; many proteins often co-occur in metal-containing fractions. Additionally, this method requires huge volumes of biomass and is both time-consuming and complex, complicating the reconstruction of the expressed metalloproteome in cells. The techniques and challenges for metalloproteomics have been discussed in detail in previously published review articles (and the references therein).^17^

In this study, we determined the trace metal utilization (the metallome) of different phytoplankton from their expressed proteome via a protein modeling and homologue identification approach, and then compared the resulting metallome with the metal quota data measured by the traditional inductively coupled plasma mass spectrometry (ICP-MS). This approach allows the building of a link between the truly used trace elements and their biological functions in phytoplankton. We aim to fill the gap between an estimation of metals encoded in the genome and metal analysis by ICP-MS in phytoplankton, which may be more broadly applicable to infer cellular metallomes.

## Materials and Methods

### Culture Conditions

Seven strains of *G. huxleyi* (OA1, OA4, OA8, OA15, OA16, OA23, and RCC 1242) and one strain of cyanobacterium *Synechocystis sp*. (FACHB898) were cultured in this study for protein extraction and subsequent ICP-MS analysis. The 6 OA strains of *G. huxleyi* were previously isolated from 3 research cruise trips.^18^ The OA1 and OA16 strains, which were also used for proteomic analysis, were originally from the North Sea and Southern Ocean, respectively. The RCC1242 strain was obtained from the Roscoff Culture Collection, France. The cyanobacterium *Synechocystis* strain was obtained from the Freshwater Algae Culture Collection at the Institute of Hydrobiology (FACHB), National Aquatic Biological Resource Center. To compare metal utilization strategies and metalloprotein expression across different strains under identical, controlled conditions, all *G. huxleyi* strains were cultured in Aquil* medium (recipe available from https://ncma.bigelow.org/algae-media-recipes) modified from ref. 19 and were kept at 20 °C at 150 μmol photons m^–2^ s^–1^ PAR (photosynthetically active radiation) with a day:night photoperiod of 12:12 h. The cyanobacterium *Synechocystis* was cultured in BG11 medium and was kept at 25 °C at 50 μmol photons m^–2^ s^–1^ PAR with a day:night photoperiod of 14:10 h.

### Proteome Data Source and Metal-Binding Estimation

In this study, we determined the metallome data from the expressed proteomes of different phytoplankton (Figure 1), including chlorophytes *Ostreococcus tauri* and *Chlamydomonas reinhardtii*, the haptophyte *Geophyracapsa huxleyi*, and the cyanobacterium *Synechocystis*. The expressed proteomic data for the chlorophytes *C. reinhardtii* and *O.tauri* were from refs. 20 and 21, respectively. Data for the cyanobacterium *Synechocystis* were from ref. 22 (data from the control group were analyzed for metal content in this study). For the haptophyte *G. huxleyi* (previously known as *Emiliania huxleyi* ^23^), proteomic data from three strains were used: data for strains OA1 and OA16 were determined in this study via protein mass spectrometry (see details below), and for the strain CCMP1516, the data were extracted from Mckew et al., (2013).^24^ The amino acid sequences of all the identified proteins from these different species were submitted to the PHYRE2 protein fold recognition server to identify homologues for known protein structures.^25,26^ Sequences where protein structures matched with a confidence >95% were then searched in the MetalPDB^27^ and PDBsum^28^ for metal-binding characterization. This approach allows for analyzing proteins with a greater variety of metals than MebiPred, which focuses on annotating 11 different metals in protein sequences.^29,30^ We normalized the measured intensity of each protein peak to the total intensity of all proteins in each species and used this relative intensity of proteins to represent their approximate abundance. Both protein abundance and predicted metal stoichiometry in the protein were taken into consideration when calculating the total metal abundance for all phytoplankton species investigated in our study.

**Figure 1:**
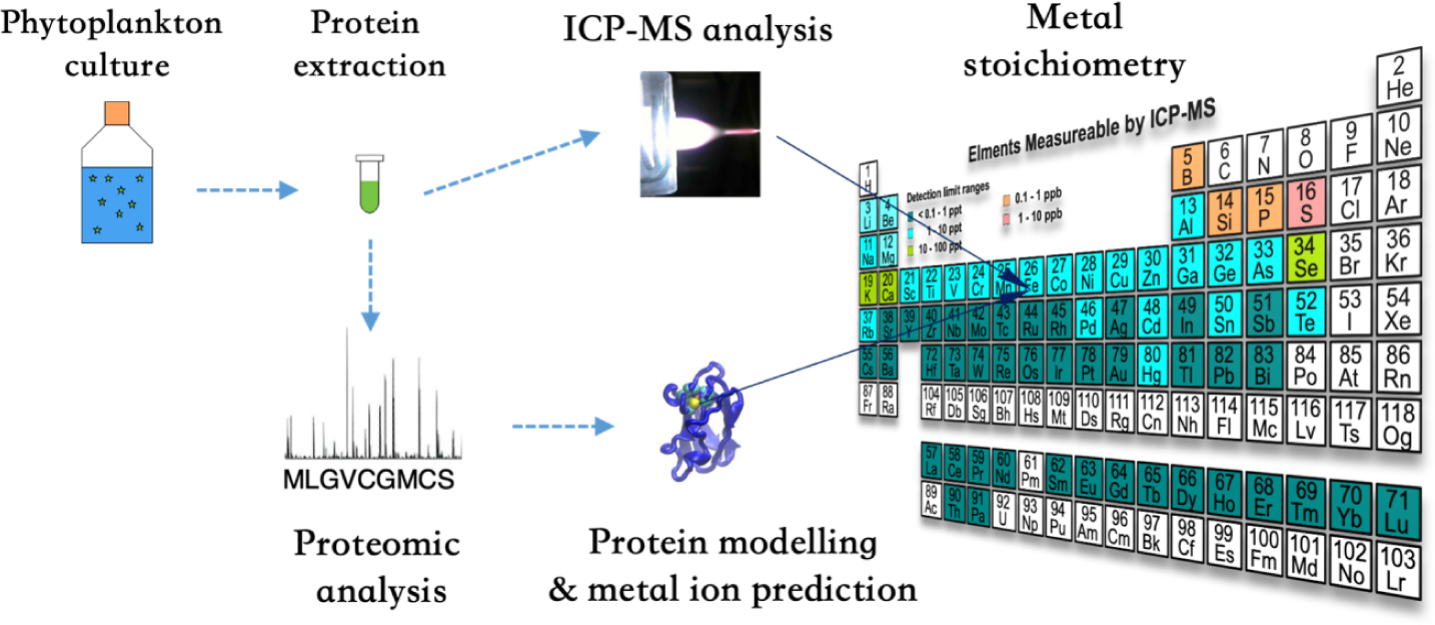
Methodology of the experiments. The metal compositions in strains *G. huxelyi* OA1 and OA16 were determined by both ICP-MS and protein modeling approaches. For the other phytoplankton species, metal compositions were only determined by the protein modeling approach.

### Protein Extraction

For protein extraction, the cells were harvested at the late exponential phase by centrifugation at 4816 RCF for 20 min and rinsed 3 times using Chelex-100 resin-cleaned synthetic ocean water (SOW) and one time with 20 mM trace metal-free Tris buffer (pH 8.0) to remove trace metals from the growth medium and those weakly bound surface metals.^31,32^ The pellets were then resuspended in extraction buffer (20 mM Tris-HCl supplemented with EDTA-free protease inhibitor (Roche), pH = 8.0) and sonicated 6 times for 30 s bursts (on ice) with a 1 min interval between sonication (Hielscher UP200S ultrasonic processor, 70% amplitude).^31^ The cell lysate was centrifuged at 10000 *g* at 4 °C for 20 min, and the supernatant containing the soluble content of the cytoplasm was passed through a precleaned 0.22 μm PTFE membrane before being subjected to trace element analysis by ICP-MS or protein digestion for LC-MS/MS measurement. It was noted that metal precipitates (e.g., Fe-oxides) may form on the cell surface during culturing and harvest, which may require intensive washing steps (e.g., using Ti- EDTA-citrate or oxalate^33^) for removal if whole-cell metal analysis is being conducted.^34–36^ To avoid uncertainties associated with potential contamination on the cell surface, we removed cell membranes from the analysis to focus on a comparison of intracellular metal quotas with those inferred from cytoplasmic proteins (and those in organelles) in this study. Since ultracentrifugation was not used in this study, debris of phytoplankton organelles was not fully removed from samples and was included in the analysis. A trace amount of cell surface membranes and associated metals may also exist in our samples.^37^ Nevertheless, since almost all of the surface membrane debris was removed by centrifugation first and then passed through the 0.22 μm filters (Supporting Information), the contamination level should be very low. The protein concentrations in the samples were measured by the Bradford assay.^38^

### ICP-MS Analysis

All preparation of samples for ICP-MS measurement was undertaken using trace metal clean methods. All plasticware used in this study, including culture flasks, pipette tips, and centrifuge tubes, was precleaned in 10% quartz-distilled HCl and thoroughly rinsed with 18 MΩ cm water in a clean laboratory. Trace elements in the filtered lysates of *G. huxleyi* (OA1, OA4, OA8, OA15, OA16, OA23, and RCC 1242) and *Synechocystis* (FACHB898) strains were measured using an ICP-MS. The detailed ICP-MS method and instrumental settings are described in Zhang et al. (2018).^31^

### Protein Sample Digestion

Protein digestion and the mass spectrometry method largely followed Zhang et al. (2024).^39^ Protein samples subjected to proteomic analysis were prepared using a Filter-Aided Sample Preparation method (FASP).^40^ Vivacon© 500 concentrators (10 kDa cutoff, Sartorius) were used for the preparation. Protein samples (20 μg from each of the triplicate samples) were loaded on the prewashed FASP filters and denatured in 8 M freshly made urea in 100 mM triethylammonium bicarbonate (TEAB) solution. The denatured proteins were then reduced with 10 mM tris (2-carboxyethyl) phosphine hydrochloride (TCEP) and alkylated with 50 mM chloroacetamide (CAA) at room temperature in the dark. The samples were then washed several times with 6 M urea in 50 mM TEAB and digested with 0.5 μg endopeptidase (LysC) for 4 h at 37 °C, and then with 0.5 μg trypsin at 37 °C after a 4-fold dilution to reduce urea concentration. The samples were collected by centrifugation, acidified by trifluoroacetic acid (TFA) to pH 3, and then desalted by using in-house-made C18 (SUPLECO, Octadecyl C18) columns.^41^ The cleaned peptide samples were dried in a centrifugal evaporator.

### Protein Mass Spectrometry

The peptides were resuspended in 5% formic acid and 5% DMSO prior to mass spectrometry. They were separated on an Ultimate 3000 UHPLC system (Thermo Fisher Scientific) and electrosprayed directly into a Q-Exactive Mass Spectrometer (Thermo Fisher Scientific) through an EASY-Spray nanoelectrospray ion source (Thermo Fisher Scientific). The peptides were trapped on a C18 PepMap100 precolumn (300 μm i.d. × 5 mm, 100 Å, Thermo Fisher Scientific) using solvent A (0.1% formic acid in water) at a pressure of 500 bar. The peptides were separated on an in-house packed analytical column (75 μm i.d. × 50 cm packed with ReproSil-Pur 120 C18-AQ, 1.9 μm, 120 Å, Dr. Maisch GmbH) using a linear gradient (length: 65 min, 15% to 35% solvent B [0.1% formic acid in acetonitrile], flow rate: 200 nL/min). The raw data were acquired on the mass spectrometer in a data-dependent mode (DDA). Full scan MS spectra were acquired in the Orbitrap (scan range 350–1500 *m*/*z*, resolution 70000, AGC target 3e6, maximum injection time 50 ms). After the MS scans, the 10 most intense peaks were selected for HCD fragmentation at 30% of normalized collision energy. HCD spectra were also acquired in the Orbitrap (resolution 17500, AGC target 5e4, maximum injection time 120 ms, isolation window 1.5 *m*/*z*) with the first fixed mass at 180 *m*/*z*. Charge exclusion was selected for unassigned and 1+ ions. The dynamic exclusion was set to 20 s.

### Proteomic Data Processing and Statistical Analysis

The resulting MS/MS data were processed using MaxQuant with the integrated Andromeda search engine (v.1.5.0.35).^42^ Tandem mass spectra were searched against the *UniProt* database (UP000013827 for *G. huxleyi*) concatenated with a reverse decoy database. The first search MS peptide tolerance was set to 50 ppm, and the match between runs function was enabled. All the other parameters in MaxQuant were set to default values.^42^ All identified protein intensities in each sample were normalized to the median intensity of all proteins to allow comparisons between samples. The average normalized intensities and standard deviations of each protein in OA1 and OA16 are listed in Table S1.

## Results and Discussion

### Trace Elements Determined from the Expressed Proteomes of Different Phytoplankton

Based on the expressed proteome sequences, we predicted metal-binding proteins and compared their distribution among various phytoplankton species (Figure 2). The inference revealed 28 different metals in *G. huxleyi*, 31 different metals in *C. reinhardtii*, and 30 different metals in *Synechocystis*. Although only 6 different metals were identified in *O. tauri* proteins, it has to be noted that the analysis for *O. tauri* was only based on the 40 most abundant proteins reported in this organism^21^ and, hence, the listed elements, including Mg, Fe, Mn, Ca, Zn, and Cu, may represent the most essential metals for the species.

**Figure 2:**
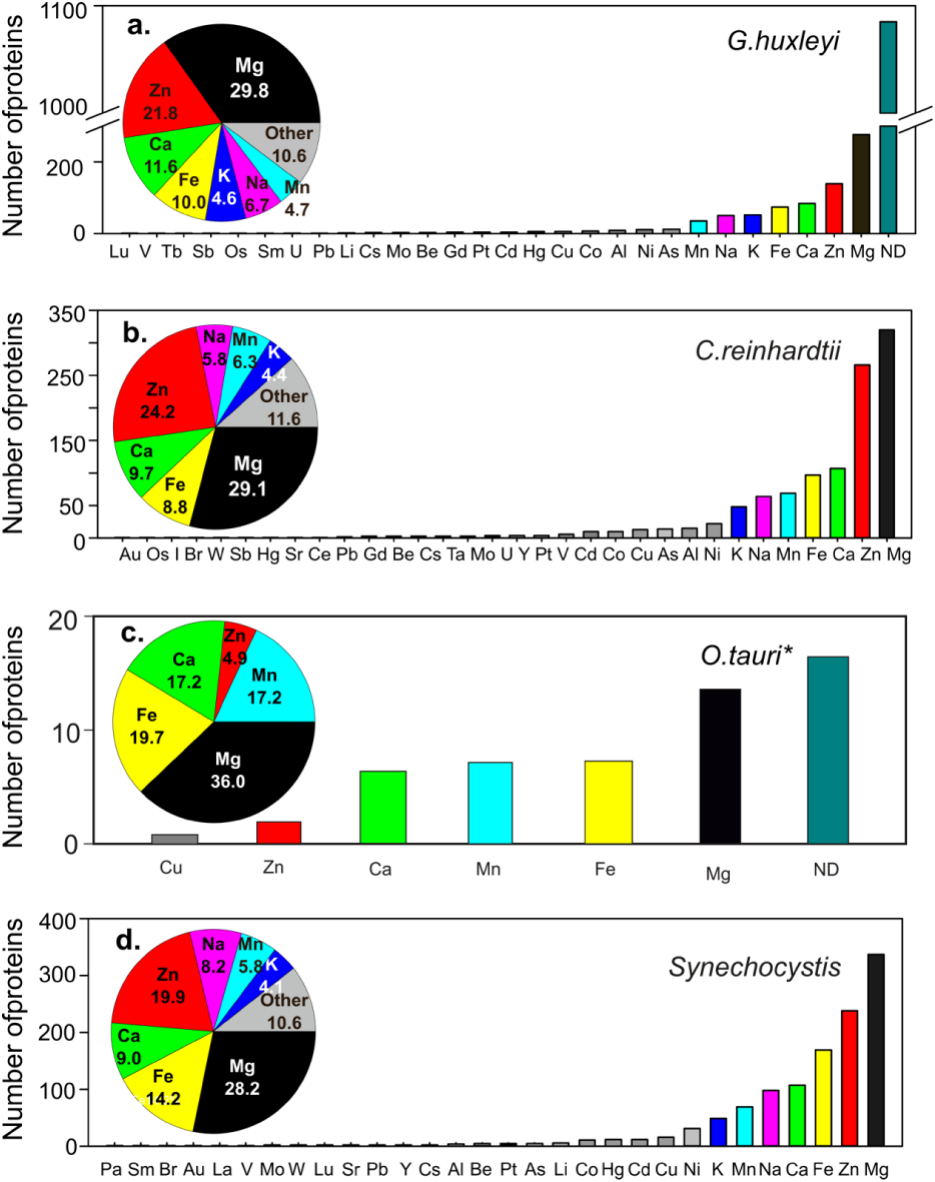
The number of metal-binding proteins identified in the expressed proteome of the haptophyte *G. huxleyi* (a), the chlorophyte *C. reinhardtii* (b) and *O. tauri*­(c), and the cyanobacteria *Synechocystis (*d). Pie charts show the percentages of metalloproteins containing specific metals. Original proteome data for *G. huxleyi* are from Mckew et al. (2013)^24^ for *C. reinhardtii* are from Hsieh et al. (2013)^77^ for *O.tauri*are from Martin et al. (2012)^21^ for *Synechocystis*are from Chen et al. (2018).^22^ *Noted that for *O. tauri*, only the 40 most abundant proteins were included in this analysis. ND: no metal binding was found in the proteins.

The number of proteins that bind with different metals varies dramatically in phytoplankton. For metalloproteins, there are variations in the binding affinities of different metals to organic molecules. For example, the affinities of divalent metals to proteins have a tendency to follow a universal order of preference, namely the Irving-Williams series (Mg^2+^ and Ca^2+^ [weakest binding] < Mn^2+^ < Fe^2+^ < Co^2+^ < Ni^2+^ < Cu^2+^ > Zn^2+^).^43^ Cells can simultaneously contain proteins having high-affinity-binding metals, such as Cu and Zn, and others that require uncompetitive metals, such as Mg and Ca. One of the least competitive metals for binding with biological chelators, Mg, is even found to be the most widely distributed metal in the metalloproteomes of various phytoplankton species in this study, supporting the observations of high concentrations in the protein fraction of cells (at millimolar concentrations^31^).

In all phytoplankton species in our analysis, the Mg-containing proteins are found to have the highest proportion in all metalloproteins identified. About 30% of the metalloproteins in different strains of phytoplankton are found to contain Mg or depend on it for function (Figure 2), suggesting the important role of Mg in phytoplankton cells. It is generally coordinated in protein complexes by ATP or ADP and has also been found to be largely distributed in the chloroplasts or thylakoids in *G. huxleyi* and *Synechocystis*.^22,24^ The essential roles of Mg in plants have been reported in various studies. Mg^2+^ is known to be the central atom of the chlorophyll molecule, and its levels in the chloroplast regulate the activity of key photosynthetic enzymes.^44^ It was reported that approximately 15–35% of total Mg is in the chloroplasts of a plant,^45^ and it also serves as an osmotically active ion in the regulation of cells^46^

For the trace elements, Zn, Fe, and Mn are the three most commonly observed metals in proteins expressed by phytoplankton (Figure 2). Excluding all nonmetal-binding proteins, in *G. huxleyi*, 21.8%, 10%, and 4.7% of the expressed metalloproteins are found to contain Zn, Fe, and Mn, respectively. In *C. reinhardtii*, similar proportions of metalloproteins were found: 24.2%, 8.8%, and 6.3% containing Zn, Fe, and Mn, respectively. The cyanobacterium *Synechocystis* has a higher percentage of Fe-containing proteins (14.2%) and a lower percentage of Zn-containing proteins (19.9%) in its expressed metalloproteome, compared to the eukaryotic phytoplankton *G. huxleyi* and *C. reinhardtii* (Figure 2). The relative sizes of expressed metalloproteomes in all species are much larger than their hypothetical sizes determined in previous comparative genomic studies.^5,14,47–49^ For example, the percentage of Zn-metalloproteins was found to constitute 2–8% and Fe-metalloproteins ∼1% of the genome-derived proteome of phytoplankton,^5^ but in the expressed proteins, Zn-containing proteins accounted for about 10%, and Fe proteins 4–7% of all proteins identified in phytoplankton (Table 1). The general requirements for major metals appear to be similar across the different species investigated in this study (Figure 2), although the percentages vary.

**Table 1:** Numbers of Zn, Fe, and Mn Metalloproteins Identified in Different Species of Phytoplankton. The Percentages of Metalloproteins in the Total Proteins Identified are Listed in the Brackets^a^

	*G. huxleyi*	*C. reinhardii*	*Synechocystis*
Total proteins	2798 (100%)	2399 (100%)	1652 (100%)
Total metalloproteins	1035 (37.0%)	868 (36.2%)	645(39.0%)
Zn-containing proteins	298 (10.6%)	266 (11.1%)	164 (9.9%)
Fe-containing proteins	137 (4.9%)	97 (4.0%)	125 (7.6%)
Mn-containing proteins	64 (2.3%)	69 (2.9%)	53 (3.2%)
Cu-containing proteins	19 (0.7%)	13 (0.5%)	12 (0.7%)

a The Original Proteome Data Were From Mckew et al., 2013 (*G. huxleyi*),^24^ Hsieh et al ., 2013 (*C. reinhardtii*),^77^ and Chen et al., 2018 (*Synechocystis*).^22^

Although a limited number of species were investigated here, the difference in the percentage of metal-binding in the expressed proteome of these species might be a reflection of their metal requirements, as cyanobacteria, in general, require less Zn but more Fe than eukaryotic phytoplankton.^5,14,50^ Cyanobacteria are believed to have evolved at least 2.75 billion years ago, and the eukaryotes *G. huxleyi* and *C. reinhardtii* evolved much later.^51^ The relationship between metal requirements in cyanobacteria and eukaryotic phytoplankton, determined from the expressed proteome, agrees well with the bioinorganic chemistry of the ocean, where soluble Fe concentration was much higher than in modern-day seawater, and soluble Zn concentration was much lower (e.g., Saito et al., 2003 ^52^). However, it is also worth noting that the difference in the percentage of Zn-containing proteins between the expressed proteomes of cyanobacteria and eukaryotic phytoplankton is less notable than that in their predicted proteomes.^5,14,47,48^ The predicted differences are largely due to the high abundance of zinc finger domains in eukaryotes,^48^ while in expressed proteomes, Zn is essential as a cofactor in many enzymes, such as carbonic anhydrase for carbon fixation, in both groups. A number of Zn-binding proteins were also identified in the cyanobacterium *Synechococcus sp*. WH8102, even under extremely low Zn concentrations.^53^ The strain was also shown to maintain Zn homeostasis by efficient Zn uptake at low Zn concentrations, underscoring the importance of investigating expressed proteomes for a more accurate understanding of metal utilization strategies in phytoplankton.

### Comparison Between Metallome Measured Directly and Reconstructed from Proteins

A large proportion of metals, such as Fe, Mn, and Cu, are found in membrane proteins,and may contribute significantly to total cellular quotas.^54,55^ However, for method development purpose, we endeavored to remove the cell membranes from our samples to focus mostly on the analysis of intracellular metal quotas and metalloproteins in the cytosol fraction in this study. We note that our analysis includes some membrane-bound proteins likely derived from organelles or surface membranes disrupted during sonication.

The derivation of cellular metal composition, as determined by two different methods, is compared in Figure 3. The abundance of metal in the proteins is determined as the relative abundance of the protein multiplied by the number of metal ions needed for the specific protein. The abundance of the proteins was normalized to ensure that the total intensities from each replicate measurement for the same species were similar. Since the proteomic data used here were generated from different studies using different instruments, we cannot quantify or compare the absolute biomass of proteins used for each species. Therefore, we cannot compare the absolute trace metal abundance between species solely from metal inferences of expressed proteomes. Nevertheless, we can estimate the relative abundance of different metals in the same species. For the elements that are both measured by the ICP-MS and identified in the proteome, there are generally good correlations (*r* ^2^ = 0.81 in Figure 3a and *r* ^2^ = 0.74 in Figure 3b) between the two approaches. This indicates that the metal compositions determined from the protein modeling may be reliable and, therefore, can reflect the metal usage in different phytoplankton. This would provide a level of assurance to further investigate the metal binding associated with various proteins, thereby deepening our understanding of the roles that different metals play in phytoplankton.

**Figure 3:**
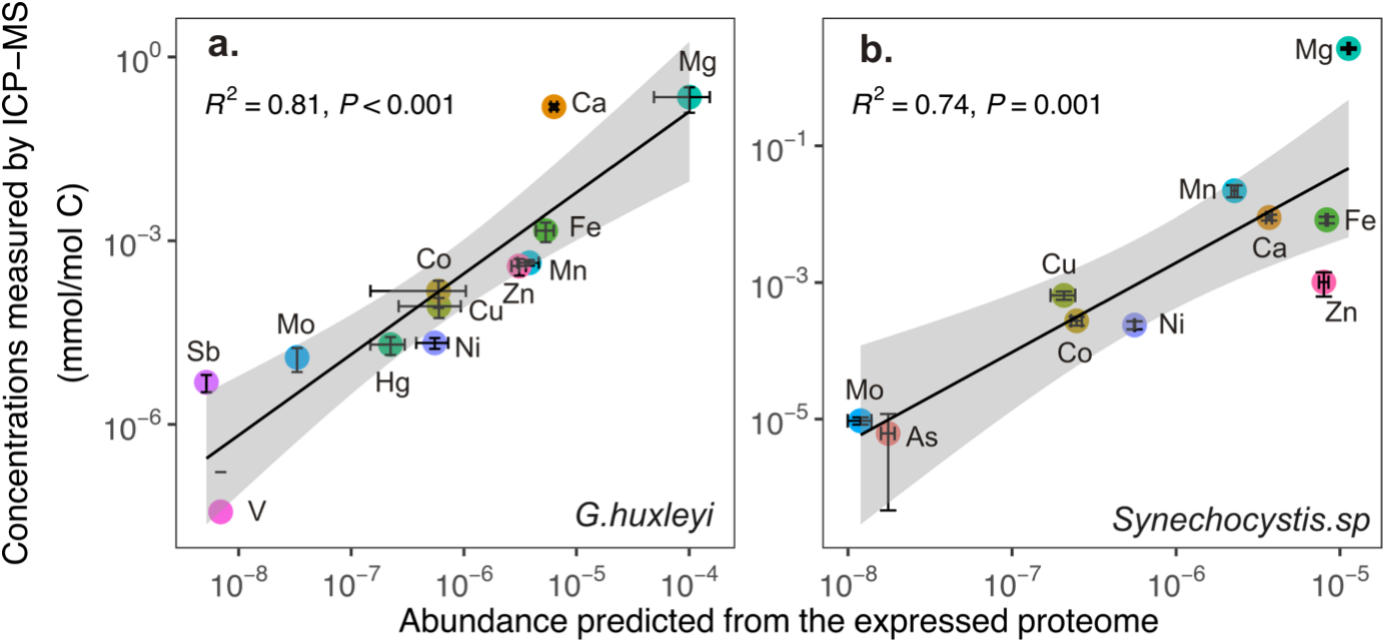
Relationship between the abundance of metals determined by ICP-MS analysis (normalized to C, mmol/mol C) and metal abundance determined from the metalloproteome analysis (the unit is arbitrary). To account for strain variations, the ICP-MS data for the cellular metal content of *G. huxleyi* are average values from 7 *G. huxleyi* strains isolated from different areas of the ocean, and the proteome-derived metal composition data are average values calculated from 2 of those *G. huxleyi* strains (OA1 and OA16) isolated previously, and data from Mckew et al., 2013.^24^ The ICP-MS data for *Synechocystis* are from *Synechocystis* sp. FACHB898 measured in this study, and the proteome data are calculated from Chen et al., 2018.^22^ Noted that the abundance of metals from each proteome was calculated by using the metal stoichiometry multiplied by the relative abundance of proteins, which provides only a relative relationship of different trace metals, not absolute abundance. The symbols represent the mean values, and the error bars indicate standard deviations.

Compared to the other metals investigated in this study, Ca and Mg appear to have a much higher relative abundance in the ICP-MS analysis than in the proteomic analysis of *G. huxleyi* and *Synechocystis* (Figure 3). These two elements are essential macronutrients for phytoplankton and plants. Ca is the most prominent secondary messenger that plays a crucial role in response to extracellular stimuli in all eukaryotes. Ca^2+^ fluxes are transported via Ca channels and transporters, such as Ca-ATPase, regulating a series of important biological functions, including mineral uptake and utilization, K^+^ homeostasis adjustment, and regulatory network activity in response to P or N deficiency.^56^ Ca is not always incorporated into protein structures. The Ca abundance determined from the proteins in this study, therefore, is much lower than the actual Ca needed intracellularly. Similarly, the intracellular Mg^2+^ and K^+^ abundances should also be higher than those determined from the protein structures. Free Mg^2+^ and K^+^ are known to regulate cation–anion balance and serve as osmotically active ions in the regulation of cells^46^ but free metal ion concentrations cannot be determined through the approach used in this study. Thus, it is not surprising to see much higher relative abundances of Ca and Mg in ICP-MS analysis than in proteomic analysis (Figure 3).

Trace metals, such as Fe, Mn, Zn, and Cu, are essential micronutrients for phytoplankton. Their distributions generally match between the two sets of analyses, although Mo concentrations appear to be higher in both species according to the ICP-MS measurements compared to those predicted from the proteome. This discrepancy may stem from our limited understanding of Mo-containing proteins, as only a few Mo-proteins have been identified in both species, potentially underestimating use. Additionally, the higher Mo levels observed in the ICP-MS analysis could be attributed to the strong binding affinity of Mo with sulfur,^57–59^ leading to its mistaken uptake by cells and subsequent storage in thiol-containing proteins intracellularly.

We also identified several unusual metals, such as Sb, As, Hg, V, and W, in both the ICP-MS and proteomic analyses. These metals are not components in the Aquil* recipe but are contaminants introduced into the cultures or samples prior to ICP-MS analysis, such as via impurities in salts that were used to prepare the growth medium and were not fully removed by the Chelex-100 resins. Once in the media, these metals may be taken up by cells and accumulate to a level that could be detected by ICP-MS. Meanwhile, some of the unusual metals could also have certain biological functions that require further exploration.^15^ While some of these elements are known to serve as metal cofactors in various proteins across different organisms,^60,61^ their biological roles remain largely unknown, particularly in marine phytoplankton. The presence of these elements in phytoplankton cells and protein structures suggests they may have potential utility for life in the ocean and warrants further investigation to validate their functions. We have listed the unusual proteins from *G. huxleyi* that may contain these metals in Table S3, which are subjects for future study. However, the deliberate addition of some unusual metals in protein structural studies raises questions about their natural occurrence and abundance. Nevertheless, the identification of these metals in cell lysates and native proteins^15,62^ provides some evidence to suggest a biological relevance, despite their enigmatic functions. This highlights a clear need for further investigation, validation, and characterization of these atypical metalloproteins in organisms from various environments.

### Can Metalloprotein Data Reveal Metal Requirements in Strains That Adapted to Different Environments?

To better understand trace metal utilization in phytoplankton, we specifically compared the trace metal compositions in two different *G. huxleyi* strains, OA1 and OA16, that were isolated from two different environments (Figures 4 and 5). OA1 was isolated from the North Sea, while OA16 was isolated from the Southern Ocean.^18^ Given that the two strains originated from distinct regions of the ocean, they may have already adapted to their specific environments, resulting in the evolution of different metal uptake and utilization strategies. We investigated the trace metal composition in OA1 and OA16 via ICP-MS and also analyzed the trace metal-containing proteins in their expressed proteomes (Table S1, Figure 4). To better compare data from these two different analyses, we presented both data as the ratio of OA16 to OA1 (OA16/OA1) in Figure 5, i.e., the ratio of trace metal concentrations in OA16 relative to OA1, compared to the ratio of trace metals in the expressed proteome of OA16 relative to those in OA1.

**Figure 4:**
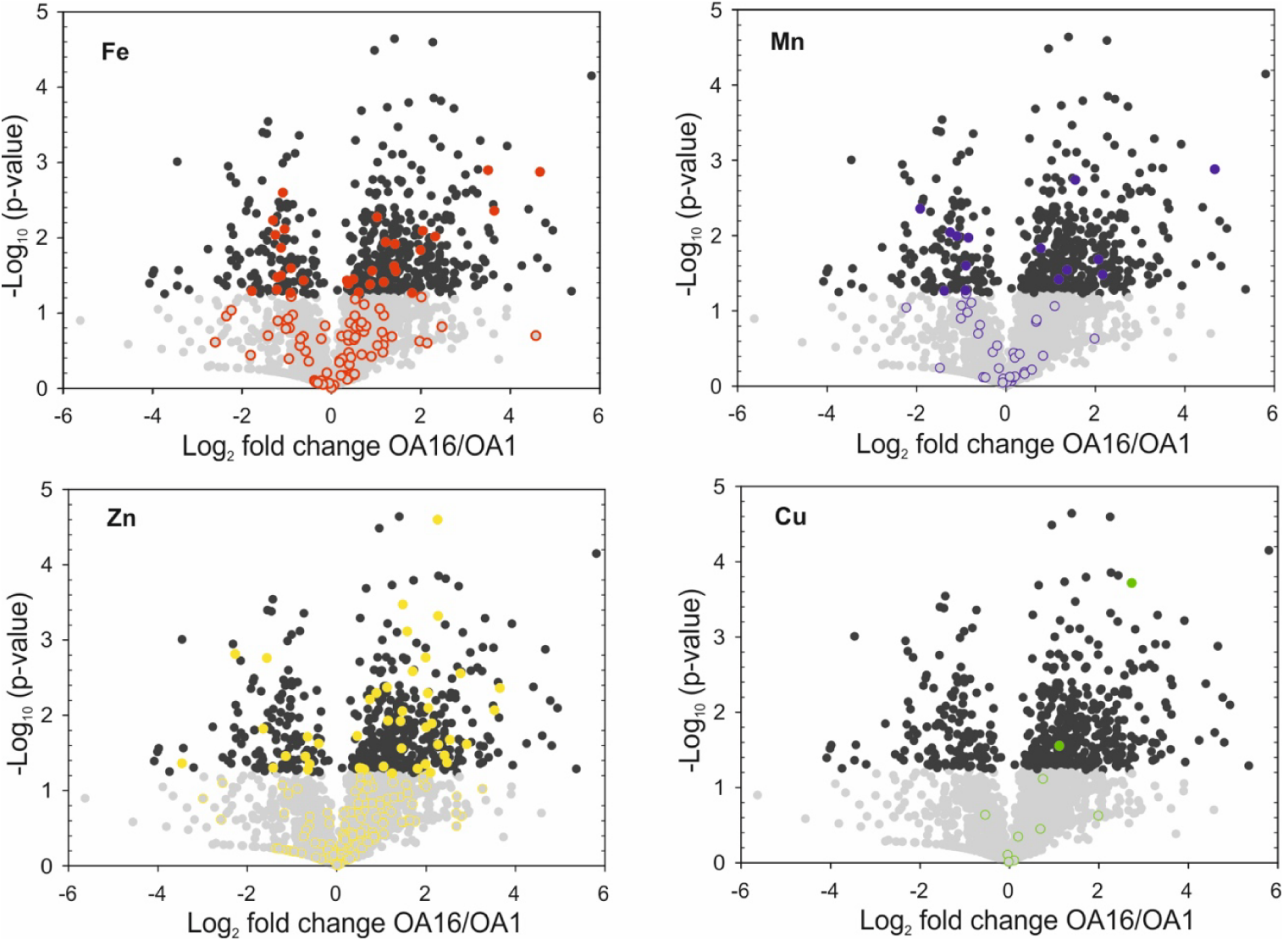
Volcano plots show that the expression of proteins containing different trace metals was significantly different in OA16 and OA1. For Fe, Mn, and Zn, various proteins were upregulated or downregulated in OA16, while for Cu, almost all proteins were upregulated in OA16. The open circles indicate metal-binding proteins that did not show significant differences between OA1 and OA16, while the filled symbols represent those that are significantly different between the two strains.

**Figure 5:**
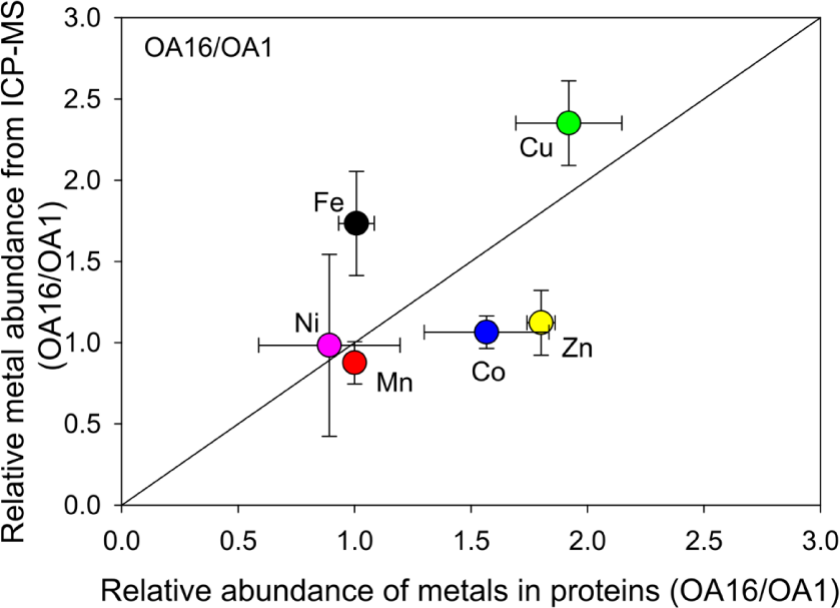
Relative relationship between trace metals determined in OA16/OA1 by ICP-MS analysis and those determined from theoretical metalloprotein analysis. The symbols represent the mean values of the ratios, and the error bars indicate the standard deviations.

The strain OA16 was isolated from the Southern Ocean, a region recognized for its Fe limitation. Consequently, these strains may have evolved to utilize Fe more efficiently. They might possess advanced strategies for transporting and storing Fe,^63^ develop protein complex structures to use less Fe to achieve the same enzymatic efficiency,^64^ or utilize other metals to substitute the biological function of Fe,^67^ adapting to the limited availability in their native environment. The Fe content in OA16 was found to be much higher than OA1 in the ICP-MS measurements (OA16/OA1 > 1, Figure 5). Higher Fe concentrations were also found in other *G. huxleyi* strains isolated from the Southern Ocean, but grown in culture conditions, compared to those isolated from the North Sea.^31^ However, in the metalloprotein analysis, the expression levels of Fe-containing proteins in both OA1 and OA16 are comparable (OA16/OA1 ≈1), suggesting higher Fe measured in OA16 may be due to a luxury uptake, i.e., OA16 has taken up more Fe than they actually need for metabolism, rather than due to a significant change in the total Fe proteins expressed in this strain (Figure 5). This further indicates that OA16 has a higher affinity for Fe uptake and storage ability intracellularly than OA1. Indeed, a higher abundance of potential metal-transporting proteins was found in OA16 compared to OA1 (Table S2). Specifically, some of the cation-transporting ATPase and vesicle-transporting proteins were detected only in OA16, indicating more transporting pathways may be employed by the species for iron uptake (Table S2). Higher concentrations of Cu were also found in OA16 than OA1 in both ICP-MS measurements (OA16/OA1 > 2, *p* < 0.05) and metalloprotein analysis (OA16/OA1 ≈ 2, *p* < 0.05, Figure 5). Such a difference may be partially due to a luxury Cu uptake but could also result from an increase in the total number of copper proteins in OA16. Most of the copper proteins identified in *G. huxleyi* were found to be upregulated in OA16 compared to OA1 (Figure 4). These copper proteins identified in this study are generally responsible for electron transfer and oxidoreductase activity, and the increase of copper utilization in *G. huxleyi* strain isolated from the Southern Ocean may reflect their strategy to deal with potential Fe limitation in the region, even if Fe was sufficiently supplied, as in our study, to support their growth. The use of copper proteins to replace the function of Fe proteins was also reported for an oceanic diatom *Thalassisira oceanica* ^66^ and the model algae *Chlamydomonas*.^65^

The total expression levels of Ni- and Mn-containing proteins in OA16 and OA1 were generally comparable (OA16/OA1 ≈ 1, Figure 5), even though specific metalloprotein expression appears significantly different (Figure 4). The Mn and Ni concentrations in OA1 and OA16 proteins from ICP-MS analysis were also comparable, indicating that the concentrations of these elements may reflect their requirements and utilization of metals in functional proteins. Although Co-proteins appear to be overexpressed in OA16 (OA16/OA1 > 1), statistical analysis (Student’s *t*-test) indicates no significant difference between the total Co contents reconstructed from the two strains’ expressed proteomes (*p* > 0.05), largely due to the uncertainties associated with Co-protein abundances, which highly depend on the accuracy of Co-binding predictions for proteins. It must be noted that many phytoplankton can use Zn and Co interchangeably,^67–70^ leading to uncertainties in predicting the *in situ* metals in the protein metal-binding sites. Nevertheless, such inter-replacement and trace metal substitution were mostly found when one metal was limited,^71^ while in our study, the metal concentrations were sufficient to support the growth of the phytoplankton. The ICP-MS analysis also indicates that the concentrations of Co were comparable in OA1 and OA16 (OA16/OA1 ≈ 1), and so the two data sets generally align with each other.

Zn was shown to be significantly more abundant in the proteome of *G. huxleyi*OA16 compared to OA1 (*p* < 0.05), while Zn concentrations measured by ICP-MS were comparable for the two strains. If Zn determined from the *G. huxleyi* expressed proteome represents the requirements of Zn by the strains, i.e., OA16 has a higher requirement for Zn than OA1, similar Zn concentrations found in the two strains might suggest a more luxurious uptake of Zn in the North Sea isolate OA1 than in the Southern Ocean strain OA16. However, *G. huxleyi* has been reported to have a relatively low requirement for Zn.^5,50^ The sufficient availability of Zn in the North Sea and Southern Ocean is unlikely to induce any Zn limitation in these strains. Therefore, the same Zn concentrations found in the two strains in this study likely reflect the strains’ affinity for transporting Zn, which is in proportion to external Zn availability. Eukaryotic phytoplankton are known to possess a complex and efficient Zn uptake system,^72^ which includes multiple ZIP (Zrt, Irt-like) family proteins that play pivotal roles in Zn transport.^73^ The ZIP family of transporters was found to be regulated by external metal availability and is reported to be actively involved in Zn homeostasis. *G. huxleyi* was found to possess many ZIP family transporters that have high thiol-containing amino acids encoded in the genome,^5^ suggesting that the species might have a good ability to take up Zn and maintain Zn homeostasis. Therefore, both *G. huxleyi* strains studied here managed to take up a similar amount of Zn intracellularly, as we have provided them with the same growth environment and medium composition.

Zn-containing proteins in both *G. huxleyi* strains were primarily associated with functions such as photosynthetic carbon fixation, gene regulation, transcription, and translation. Research indicates that Southern Ocean phytoplankton enhance their Zn uptake to meet increased metabolic demands in response to potential CO_2_ limitation in low *p*CO_2_ regions.^74^ A high intracellular Zn demand may be a signature for the Southern Ocean strains, including OA16, which we investigated in this study. Notably, more types of Zn-containing proteins were identified in OA16 compared to OA1 (Table S4), although their functions remain poorly annotated based on current knowledge. Furthermore, several functions of Zn appear significantly upregulated in OA16 (Figure 4, Table S5). It is likely that if environmental Zn limitations occur, strain OA16 might be more adversely affected than strain OA1, which requires less Zn for transcription and metabolic functions.

Overall, despite being cultured in the same medium, the *G. huxleyi* strains displayed distinct intracellular Fe and Cu metal quotas and metalloprotein profiles, highlighting inherent variability likely shaped by their evolutionary history. Our analysis demonstrates that investigating metalloprotein expressions in phytoplankton can provide a much deeper understanding of the physiological and metabolic roles of different trace metals. Moving forward, this approach could be used to explore phytoplankton metalloproteomes under various conditions, enhancing our understanding of how metal demands may change in natural environments.

### Limitations and Future Mitigations

Our sample preparation aimed to remove cell surface membranes and focus on intracellular components for ICP-MS and proteomic analysis. An additional analysis was performed to evaluate the efficiency of the removal of cell surface membrane fragments in our usual sample methodology. We ran parallel measurements of samples with and without an additional ultracentrifugation step and measured their phosphorus concentrations after digestion (Supporting Information). Since phosphorus is a major component in the membrane, if a significant amount of additional phosphorus was removed by this extra ultracentrifugation step, then it is reasonable to suggest that our methodology had been insufficient to remove the contribution of cell membrane debris, even after centrifugation and filtering steps. However, in our study, we observed quite the reverse. We observed that over 95% of the phosphorus content of samples was preserved in the samples even after this additional ultracentrifugation step (99.2 ± 6.4%). This suggests that the contamination of cell surface membrane debris was insignificant to the final sample analysis by using our typical centrifugation and filtering protocol (Figure S1). Furthermore, the concentrations of most trace metals, including Ba and V, which are known to easily adsorb to FeO_*x*_,^34^ were comparable before and after ultracentrifugation (∼100%, Figure S2), suggesting FeO_*x*_ contamination levels were very low, if not negligible. However, we did observe a decrease of Fe concentrations after ultracentrifugation (53.2 ± 8.7%, Figure S2). While this loss is consistent with the removal of Fe-rich organelle components and membrane debris (e.g., chloroplasts and mitochondria^75,76^ ) during ultracentrifugation, we cannot rule out the coremoval of trace FeO_*x*_ solids that may have adsorbed to surface membrane fragments since we did not use a Ti- EDTA-citrate or oxalate wash.^33^ This introduces uncertainty in our Fe quantification. Therefore, future methods can mitigate these potential limitations via a more rigorous washing step before cell lysis, to ensure minimal FeO_*x*_ contamination in sample preparations, which could further enhance the accuracy of metal concentration analysis.^33^

## Supplementary Material





## Notes

Additional details for cell lysate ultracentrifugation experiments, including supplementary methods and results (PDF)
All proteins identified in the *Geophyracapsa huxleyi* OA1 and OA16 (Table S1); potential metal transporting proteins in the *Geophyracapsa huxleyi* OA1 and OA16 (Table S2); *G. huxleyi* proteins identified as containing unusual metal-binding cofactors (Table S3); potential Zn-containing proteins that were only detected in *G.huxleyi* OA16. (Table S4); the top 10 functions of Zn-containing proteins that showed differential expression between OA16 and OA1 (Table S5); Potential phytochelatin synthesaes in *G. huxleyi* (Table S6); metal concentrations in *Synechocystis* and *G. huxleyi* (Table S7) (XLSX)
