# Supplementary material for: Unlocking Phytoplankton Metallomes with Comparative Analysis of Metal Quotas, Quantitative Proteomics, and Inferred Metalloproteomes
Source: Environ Sci Technol. 2025 Dec 12;59(50):27367–77. doi: 10.1021/acs.est.5c11233 (PMC12750528; doi:10.1021/acs.est.5c11233)
Supplement: Supplementary file 1 [file es5c11233_si_001.pdf]

Supplementary materials for *Unlocking Phytoplankton Metallomes with Comparative Analysis of Metal Quotas, Quantitative Proteomics, and Inferred Metalloproteomes*

Qiong Zhang[1,2,3*], Jiayou Ge[1], Fengjie Liu[4], Shabaz Mohammed[5,6], Kedong Yin[3,7], Rosalind E. M. Rickaby[8]

[1] Department of Ocean Science, The Hong Kong University of Science and Technology, Clear Water Bay, Hong Kong SAR

[2] Center for Ocean Research in Hong Kong and Macau (CORE), The Hong Kong University of Science and Technology, Clear Water Bay, Hong Kong SAR

[3] Southern Marine Science and Engineering Guangdong Laboratory (Zhuhai), Zhuhai, 519000 China

[4] Grantham Institute-Climate Change and the Environment and Department of Life Sciences, Imperial College London, Exhitition Road, London, SW7 2AZ, United Kingdom

[5] Department of Biochemistry, University of Oxford, South Parks Road. OX1 3QU, United Kingdom

[6] Department of Chemistry, University of Oxford, South Parks Road, OX1 3TA, United Kingdom

[7] School of Marine Science, Sun Yat-Sen University, Zhuhai, 519000, China

[8] Department of Earth Sciences, University of Oxford, South Parks Road. OX1 3AN, United Kingdom

*Corresponding author: Qiong Zhang (qiongz@ust.hk)

Supplementary methods

To evaluate the efficiency in removing cell surface membrane debris via centrifugation and then filtering through 0.22 um filters, we conducted an analysis of phosphorus, the major component of the lipid bilayers of the cell surface membrane, before and after a subsequent ultracentrifugation step.

Four biological replicates were prepared for this analysis. For each culture replicate, the cells were cultured and harvested in the same way described in Method for *G. huxleyi.* The cells were disrupted by sonication, centrifuged, and then passed through the 0.22 μm filters. Each cell lysate was then evenly subsampled into two parts. One was digested directly. The other underwent a subsequent ultracentrifugation at 10,000 g for one hour. The solution after ultracentrifugation was also digested. The two subsamples were measured in the same way by ICP-MS.

Supplementary Figures

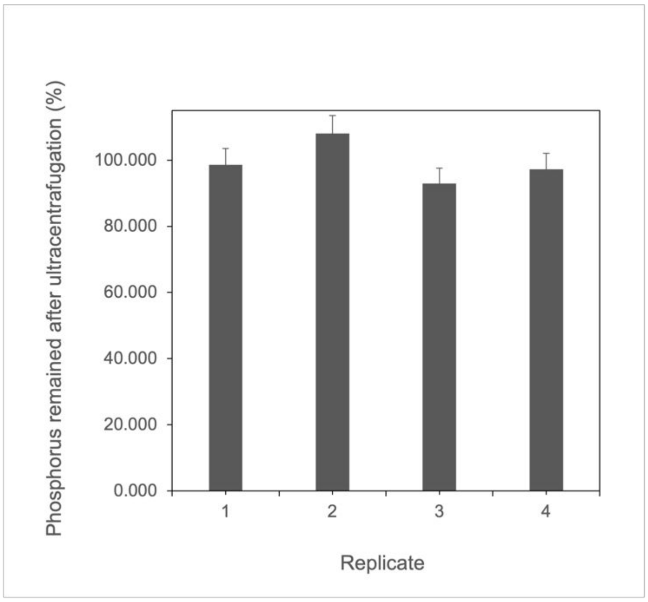

Figure S1. The ratio of phosphorus concentrations after ultracentrifugation to their concentrations without ultracentrifugation. Each bar represents one biological replicate of the sample. The error bars indicate the relative standard deviations of the measurement.

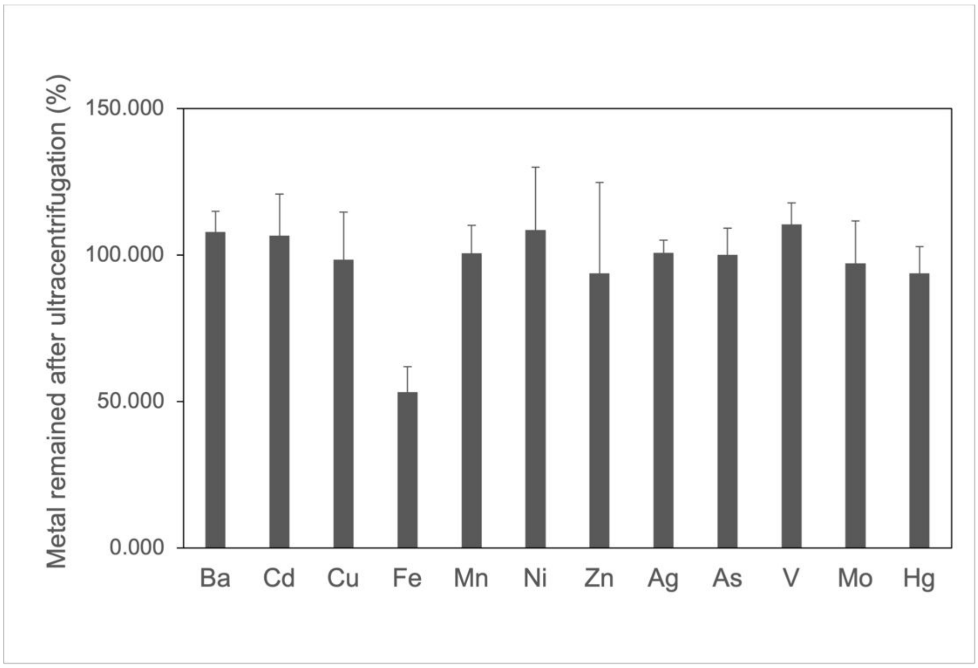

Figure S2. The ratio of metal concentrations after ultracentrifugation to their concentrations without ultracentrifugation. The bars represent the mean values of the ratios of the four replicates. The error bars indicate the standard deviations of the ratios among the four replicates.
